# Entwicklung von Qualitätsstandards für die Versorgung von Patient*innen mit rheumatoider Arthritis zur Anwendung in Deutschland

**DOI:** 10.1007/s00393-021-01093-1

**Published:** 2021-10-15

**Authors:** U. Kiltz, V. Buschhorn-Milberger, K. Albrecht, H.-J. Lakomek, H.-M. Lorenz, M. Rudwaleit, M. Schneider, H. Schulze-Koops, M. Aringer, M. I. Hasenbring, P. Herzer, U. von Hinüber, K. Krüger, A. Lauterbach, B. Manger, R. Oltman, F. Schuch, R. Schmale-Grede, S. Späthling-Mestekemper, S. Zinke, J. Braun

**Affiliations:** 1 Rheumazentrum Ruhrgebiet, Claudiusstr. 45, 44649 Herne, Deutschland; 2 Ruhr-Universität Bochum, Bochum, Deutschland; 3 Programmbereich Epidemiologie, Deutsches Rheuma-Forschungszentrum (DRFZ), Berlin, Deutschland; 4 Johannes-Wesling-Klinikum Minden, Universitätsklinik für Geriatrie, Minden, Deutschland; 5 Sektion Rheumatologie, Medizinische Klinik V, Universitätsklinikum Heidelberg, Universität Heidelberg, Heidelberg, Deutschland; 6 Universitätsklinik für Innere Medizin und Rheumatologie, Klinikum Bielefeld Rosenhöhe, Universität Bielefeld, Bielefeld, Deutschland; 7 Poliklinik, Funktionsbereich und Hiller Forschungszentrum für Rheumatologie, Universitätsklinikum Düsseldorf, Heinrich-Heine-Universität Düsseldorf, Düsseldorf, Deutschland; 8 Sektion Rheumatologie und Klinische Immunologie, Medizinische Klinik und Poliklinik IV, LMU-Klinikum München, Ludwig-Maximilians-Universität München, München, Deutschland; 9 Medizinische Klinik und Poliklinik III, Rheumatologie, Universitätsklinikum Carl Gustav Carus, Technische Universität Dresden, Dresden, Deutschland; 10 Abteilung für Medizinische Psychologie und Medizinische Soziologie, Ruhr-Universität Bochum, Bochum, Deutschland; 11 Medicover München MVZ, München, Deutschland; 12 Praxis für Rheumatologie und Osteologie, Hildesheim, Deutschland; 13 Rheumatologisches Praxiszentrum St. Bonifatius, München, Deutschland; 14 Physiotherapieschule Friedrichsheim, Friedrichsheim, Deutschland; 15 Medizinische Klinik 3 Rheumatologie und Immunologie, Universitätsklinikum, Friedrich-Alexander-Universität Erlangen/Nürnberg, Erlangen, Deutschland; 16 Hochschule für Gesundheit Bochum, Bochum, Deutschland; 17 Rheumatologische Schwerpunktpraxis Erlangen, Erlangen, Deutschland; 18 Deutsche Rheuma-Liga Bundesverband e. V., Bonn, Deutschland; 19 Rheumapraxis München, München, Deutschland; 20 Rheumatologische Schwerpunktpraxis Zinke, Berlin, Deutschland; 21 Bundesverband Deutscher Rheumatologen e. V. (BDRh), Grünwald, Deutschland

**Keywords:** Rheumatoide Arthritis, Versorgungsqualität, Qualitätsstandards, Versorgungslücken, Rheumatoid arthritis, Quality of care, Quality standards, Care gaps

## Abstract

Trotz einer qualitativ und strukturell guten Versorgung von Patient*innen mit rheumatoider Arthritis (RA) in Deutschland bestehen weiterhin potenziell behebbare Defizite in der Qualität der Versorgung. Aus diesem Grund hat die Deutsche Gesellschaft für Rheumatologie (DGRh) eine Expert*innengruppe, in der verschiedene Interessengruppen vertreten waren, beauftragt, nationale Qualitätsstandards (QS) mit dem Ziel zu entwickeln, die rheumatologische Versorgung von Patient*innen mit RA in Deutschland qualitativ zu verbessern. QS dienen der Festlegung und quantitativen Messung guter Versorgungsqualität unter dem Vorbehalt von Relevanz und Realisierbarkeit. Als Grundlage für die Entwicklung dienten die kürzlich publizierten Standards von NICE und ASAS und eine systematische Literatursuche. Insgesamt wurden 8 hiermit erstmals veröffentlichte QS konsentiert, die als Grundlage dienen können, die Versorgungsqualität von Patient*innen mit RA in Deutschland zu messen und weiter zu optimieren.

**Zusatzmaterial online:**

Zusätzliche Informationen sind in der Online-Version dieses Artikels (10.1007/s00393-021-01093-1) enthalten.

## Abstract (en)

Despite a qualitatively and structurally good care of patients with rheumatoid arthritis (RA) in Germany, there are still potentially amendable deficits in the quality of care. For this reason, the German Society for Rheumatology (DGRh) has therefore decided to ask a group of experts including various stakeholders to develop quality standards (QS) for the care of patients with RA in order to improve the quality of care. The QS are used to determine and quantitatively measure the quality of care, subject to relevance and feasibility. The recently published NICE and ASAS standards and a systematic literature search were used as the basis for development. A total of 8 QS, now published for the first time, were approved with the intention to measure and further optimize the quality of care for patients with RA in Germany.

Die rheumatoide Arthritis (RA) ist eine meist die peripheren Gelenke von Händen und Füßen betreffende entzündlich rheumatische Erkrankung, welche unbehandelt oft zu Osteodestruktionen durch Gelenkerosionen und Gelenkfehlstellungen führt [1]. Initial treten in der Regel symmetrische Schwellungen von Gelenken zusammen mit länger anhaltender Morgensteifigkeit auf. Auch in anderen Organen wie der Lunge oder den Blutgefäßen kann es zu Manifestationen der RA kommen.

Die Versorgungsqualität, nicht nur von Patient*innen mit RA, weist trotz Entwicklung und regelmäßiger Aktualisierung evidenzbasierter Leitlinien (LL) durch die Fachgesellschaft DGRh [2, 3] nach wie vor in einigen Bereichen Defizite auf. So fand sich v. a. bei älteren RA-Patient*innen, bei RA-Patient*innen ohne laborchemischen Nachweis von Rheumafaktor sowie bei RA-Patient*innen ohne fachärztliche Betreuung eine Unterversorgung mit DMARDs, insbesondere mit Biologika (bDMARDs) [4–7]. Zusätzlich konnten eine Unterversorgung in der Verordnung von Physiotherapie [8, 9], eine eher inkonsequent angewendete Treat-to-target-Strategie [5], zu späte oder fehlende Überweisungen von RA-Verdachtsfällen [10] und die nicht seltene Versorgung durch Ärzt*innen ohne internistisch-rheumatologische Facharztkompetenz aufgezeigt werden [4]. Darüber hinaus gibt es Hinweise auf eine unzureichende Versorgung von Patient*innen mit Komorbiditäten [11]. Auch zeigen sich eine Unterversorgung der Komorbidität Osteoporose [12] und unzureichende Präventivmaßnahmen, wie z. B. Impfungen [13, 14]. Außerdem werden RA-Patienten*innen, die im Pflegeheim leben, weniger häufig von rheumatologischen Fachärzt*innen betreut und erhalten demnach seltener DMARDs [15].

Dessen ungeachtet sind durchaus auch positive Entwicklungen zu verzeichnen wie etwa die Einführung der ambulanten spezialfachärztlichen Versorgung (ASV), die Einführung von Früharthritissprechstunden, die Anerkennung der rheumatologischen Fachassistenz (RFA) durch die Bundesärztekammer, das Patientenschulungs- und Informationsprogramm der DGRh [16] und nicht zuletzt das durch den Gemeinsamen Bundesauschuss beschlossene Disease-Management-Programm RA [17].

Das Ziel der Entwicklung von Qualitätsstandards (QS) ist es, bestehende Versorgungslücken zu identifizieren und dann den vorliegenden Problemen entsprechende standardisierte Messparameter für die Verbesserung der Qualität in den betroffenen Versorgungsbereichen vorzuschlagen.

Bislang gibt es zwar internationale QS, auf nationaler Ebene wurden aber bisher noch keine QS für die Versorgung von RA-Patient*innen generiert. International wurden einige z. T. sehr umfangreiche Pakete von Qualitätsstandards und/oder -indikatoren entwickelt. Während sich die adressierten Schlüsselbereiche zur Qualitätsverbesserung bei diesen Vorschlägen häufig ähneln und zum Teil überlappen, ist es im Rahmen der Entwicklung der QS zu sehr unterschiedlichen Vorgehensweisen gekommen, d. h. eine einheitliche Methodik wurde bislang nicht verwendet. Es gibt mehrere Modelle zur Entwicklung von QS [18]. In England hat sich das National Institute for Health and Care Excellence (NICE) auf eine Methodik geeinigt [19], auf die auch bereits an anderer Stelle [20] zurückgegriffen wurde. Allen Modellen ist gemeinsam, dass gute Versorgungsqualität auf Grundlage der Arbeiten von Donabedian definiert wird [21]. Dies beinhaltet, dass bei der Beurteilung von Versorgungsqualität neben Struktur- und Prozessqualität auch Ergebnisqualität zu berücksichtigen ist. Während die Strukturqualität v. a. durch das Vorhandensein von definierten Strukturen wie etwa von geeigneten Ambulanzräumen, einem Ultraschallgerät oder der Vorhaltung eines behindertengerechten Praxiszugangs gemessen wird, bedeutet Prozessqualität die Organisation bzw. den Ablauf eines Prozesses, wie z. B. die Terminplanung einer Sprechstunde oder die Verfügbarkeit von geeignetem Praxispersonal. Struktur- und Prozessqualität sind eine wichtige Grundlage für die Ergebnisqualität, die unter anderem den Therapierfolg abbildet, wobei z. B. die Anzahl von Patient*innen, die in einem definierten Zeitraum eine Remission erreichen, gemessen werden könnte [21].

Ein erstes Projekt zur Qualitätssicherung der Versorgung von rheumatischen Erkrankungen gibt es in Deutschland bereits seit geraumer Zeit in Form des OBRA („outcome benchmarking“ in der rheumatologischen Akutversorgung) bzw. KOBRA(kontinuierliches Outcome-Benchmarking in rheumatologischen Akutkliniken)-Projekts, welches seit Jahren in mehreren Kliniken der akutstationären Versorgung durch den Verband Rheumatologischer Akutkliniken (VRA) in Kooperation mit dem Institut für angewandte Qualitätsförderung und Forschung im Gesundheitswesen (AQUA) erfolgreich durchgeführt wird [22, 23].

Hierfür wurden Qualitätsindikatoren definiert, um Versorgungsqualität und Patient*innenzufriedenheit zu messen und um zwischen verschiedenen Kliniken Vergleiche anstellen zu können (Benchmarking) – mit dem Ziel, die Versorgungsqualität zu verbessern. Die Daten werden in den Kliniken erhoben und vom unabhängigen AQUA-Institut analysiert und ausgewertet. Für die Teilnahme am Benchmarkingprozess erhalten die teilnehmenden Kliniken, wenn sie auch sonst die Qualitätsansprüche erfüllen [24], ein Zertifikat und Gütesiegel des VRA. Nachdem zunächst nur Patient*innen mit RA in das Projekt eingeschlossen wurden, wird inzwischen ein deutlich größeres Spektrum entzündlich rheumatischer Erkrankungen erfasst.

Die Deutsche Gesellschaft für Rheumatologie (DGRh) hat es sich zur Aufgabe gemacht, nationale QS zu formulieren, um die Versorgungsqualität von RA-Patient*innen in Deutschland messen und verbessern zu können. Dabei sollte der formulierte Qualitätsstandard ein wünschenswertes, jedoch auch realisierbares, Versorgungsziel repräsentieren. Das langfristige Ziel besteht darin, die Qualität in Deutschland durch messbare Qualitätskonstrukte zu dokumentieren und zu verbessern, um in Zukunft ein hohes Maß an Versorgungsqualität von RA-Patient*innen gewährleisten zu können.

## Methoden

Die Entwicklung der Qualitätsstandards für RA startete im September 2019 mit der Bildung eine Steuerungsgruppe mit ausgewählten Expert*innen mit Expertise in der Versorgung von Patient*innen mit RA (KA, JB, UK, HS‑K, H‑JL, H‑ML, MR, MS). Die Steuerungsgruppe entschied sich nach eingehender Diskussion, alle in der Behandlung der RA involvierten Interessengruppen (ambulant und stationär tätige Rheumatolog*innen, Physiotherapeut*innen, Ergotherapeut*innen, Psycholog*innen, Patient*innenvertreter*innen) aktiv in die Entwicklung der QS für RA als Arbeitsgruppe (AG) einzubeziehen. Alle eingeladenen Vertreter (Physiotherapie vertreten durch AL, Ergotherapeuten durch RO, Psychologen durch MIH, Patienten durch RSG) beteiligten sich aktiv an dem Entwicklungsprozess und waren stimmberechtigt. Nicht stimmberechtigt war VBM als Methodikerin. Die Steuerungsgruppe legte die Methodik und den Ablauf der Entwicklung der Qualitätsstandards in 5 Phasen fest (Tab. 1).

**Table 1:** 

Phase	Ziel	Methode
1	Teil 1: Identifikation von publizierten Qualitätsmessinstrumenten für RA (bezogen auf Methodik und Inhalt) Teil 2: Identifikation von relevanten Versorgungslücken	SLR
2	Identifikation der Schlüsselbereiche für die Qualitätsverbesserung	Virtuelles Treffen
3	Priorisierung der Schlüsselbereiche für die Qualitätsverbesserung	Standardisierte Online-Abfrage
4	Formulierung der Qualitätsstandards für RA	Strukturierte Konsensfindung, virtuell
5	Konsentierung der Qualitätsstandards für RA	Grad der Zustimmung, NRS 0–10

Die Entwicklung der Qualitätsstandards für RA erfolgte parallel zu der Erstellung der Qualitätsstandards für die axiale Spondyloarthritis [25].

### Phase 1 mit Erstellung der systematischen Literaturrecherche.

Beide SLR wurden in PubMed und Cochrane für den Zeitraum 01.05.2005–01.05.2020 durchgeführt (Suchterms s. Online-Zusatzmaterial). Es wurde von der Steuerungsgruppe festgelegt, dass englisch- und deutschsprachige Publikationen als Vollpublikation eingeschlossen werden konnten. Die Suche zu Qualitätsmessinstrumenten für RA fokussierte auf die Identifikation von Methodik und Inhalt. Für die Selektion von Publikationen zu Versorgungslücken wurden folgende Einschlusskriterien festgelegt: Studientyp (kontrollierte Studien/Kohortenstudien/Fall-Kontrollstudien mit einer Fallzahl ≥ 200 Patient*innen) und qualitative Studien (ohne Teilnehmerbegrenzung).

### Phase 2: Identifikation der Schlüsselbereiche für die Qualitätsverbesserung.

Schlüsselbereiche sind Bereiche, in denen es Unterschiede in der Versorgung gibt, deren Verbesserung aber realistisch ist und auch quantitativ erfasst werden kann. Auf der Grundlage der SLR erfolgte die Identifikation von Versorgungslücken. Die Schlüsselbereiche sind unter folgenden 5 Aspekten diskutiert worden: (I) Validität („Wie valide ist die wissenschaftliche Evidenz, dass es sich bei dem genannten Schlüsselbereich, um eine im klinischen Alltag relevante Domäne handelt?“), (II) Augenscheinvalidität („Wie wahrscheinlich ist es, dass eine bessere Versorgungsleistung in dem genannten Schlüsselbereich ein qualitativ hochwertigeres Gesundheitssystem widerspiegelt?“), (III) Durchführbarkeit („Wie wahrscheinlich ist es, dass die für den Schlüsselbereich erforderliche Information in Ihrem Gesundheitssystem verfügbar ist?“), (IV) Bedeutung: („Wie wichtig ist es, diesen Schlüsselbereich zu messen?“) und (V) Wahrscheinlichkeit der Verwendung („Wie wahrscheinlich ist es, dass Sie diesen Schlüsselbereich in Ihrer Praxis/Klinik verwenden oder zur Verwendung dieses Indikators ermutigen würden?“). Die AG diskutierte auf einem virtuellen Treffen die Versorgungslücken sehr intensiv und legte eine Liste mit Versorgungslücken an, die quantitativ erfasst werden können.

### Phase 3: Priorisierung der Versorgungslücken.

Die identifizierten Schlüsselbereiche wurden im Anschluss an das virtuelle Treffen per Online-Umfrage priorisiert. Ziel der Priorisierung war es, die relevanten Schlüsselbereiche zu identifizieren und die vorläufige Liste auf eine handhabbare finale Liste mit ca. 7 bis 10 Schlüsselbereichen für die Qualitätsverbesserung zu reduzieren. Die Priorisierung wurde erhoben, indem nach der Relevanz („Stimmen Sie zu, dass diese Domäne eine relevante Versorgungslücke in Deutschland darstellt?“) und nach dem Grad der Zustimmung (numerische Rating-Skala 0–10, 10 = höchste Zustimmung) gefragt wurde.

### Phase 4: Formulierung der Qualitätsstandards für RA.

Nach Festlegung der 8 Versorgungslücken erfolgte in einem virtuellen Treffen die Ausformulierung der Qualitätsstandards in Bezug auf Struktur und Prozessqualität. Ein Qualitätsstandard besteht aus 2 Bereichen: (I) der Kernaussage mit einer begleitenden Rationale, in der die Evidenz für die Aussage dargelegt wird, und (II) einem Messinstrument, in dem die Angaben zur Berechnung der Struktur- und Prozessqualität angegeben sind. Das Ziel der AG in dem virtuellen Meeting im Dezember 2020 war es, Kernaussagen zu erarbeiten und diese dann jeweils mit einer Rationale und Qualitätsmessinstrumenten zu unterfüttern. Die im Anschluss erfolgte schriftliche Ausformulierung der QS wurde mittels E‑Mail-Schriftverkehr mehrfach überarbeitet.

### Phase 5: Konsentierung der Qualitätsstandards.

Nach Ausformulierung der Qualitätsstandards wurden die Teilnehmer der AG gebeten, in einer Online-Umfrage den Grad der Zustimmung (mittels NRS 0–10, 10 stimme voll zu) anzugeben.

## Ergebnisse

### Erstellung der Qualitätsstandards

Die Analyse der einzelnen Schritte ergab folgende Ergebnisse:

#### Phase 1 mit Erstellung der systematischen Literaturrecherche

**Figure 1:**
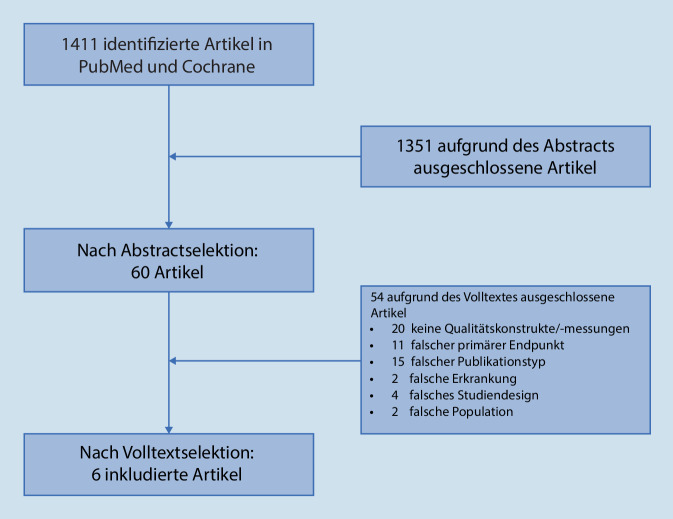

SLR Qualitätsmessinstrumente: Die einzelnen Schritte der SLR hinsichtlich bereits publizierter Qualitätsmessinstrumente listet Abb. 1 auf. Insgesamt wurden 6 Publikationen zu Qualitätsmessinstrumenten der RA identifiziert [26–31] Die ersten QS wurden 2005 in England veröffentlicht [30], gefolgt von Publikationen in den USA und Europa [27, 31]. Drei weitere Artikel beziehen sich auf Arthritiden allgemein und stellen somit keine Qualitätsmessinstrumente speziell für RA dar [32–34]. Allen Publikationen gemeinsam ist, dass es keine einheitlich verwendeten Qualitätsmessinstrumente gibt und dass die jeweils gewählte Methodik zur Erstellung der Qualitätsmessinstrumente unterschiedlich ist.

##### SLR Versorgungslücken.

**Figure 2:**
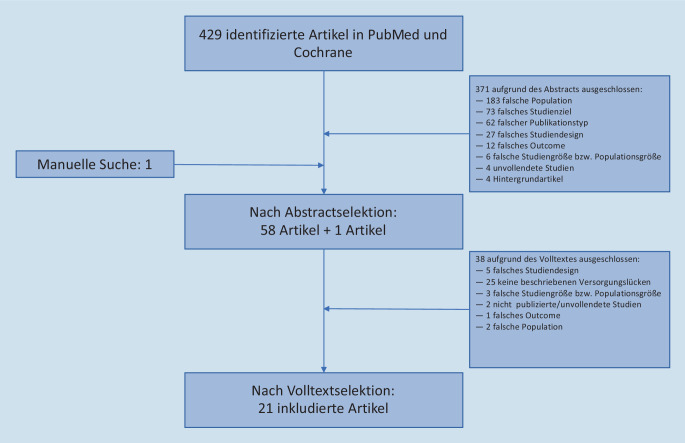

In Abb. 2 sind die einzelnen Schritte der SLR bezüglich der Versorgungslücken aufgeführt. Es wurden insgesamt 21 Studien für Versorgungslücken in den folgenden Domänen, identifiziert: Therapie (*n* = 7), Komorbidität (*n* = 7), Management (*n* = 6), Risikogruppe (*n* = 3), Überweisung (*n* = 3) und Training (*n* = 2).

#### Phase 2: Identifikation der Schlüsselbereiche für die Qualitätsverbesserung

Die AG identifizierte in einem virtuellen Treffen im Oktober 2020 insgesamt 19 Schlüsselbereiche, die quantitativ erfasst werden können (Tab. 2).

**Table 2:** 

QS Nr	Text QS	Stimmen Sie zu, dass diese Domäne eine relevante Versorgungslücke in Deutschland darstellt?	Bitte geben Sie den Grad der Zustimmung für diese Domäne an	
			Mean	SD
1	Überweisung zu dem*der Rheumatolog*in	84,6 % Ja	7,8	2,3
2	Frühzeitige Diagnose (innerhalb von 6 Wochen nach Vorstellung bei dem*der Hausarzt*ärztin)	92,3 % Ja	9,4	0,7
3	Versorgung mit einer Basistherapie bei gesicherter Diagnose	69,2 % Ja	6,3	3,0
3a	Versorgung mit einer Basistherapie bei gesicherter Diagnose: Gesamtpopulation	61,5 % Ja	6,1	3,2
3b	Versorgung mit einer Basistherapie bei gesicherter Diagnose: Populationen mit Besonderheiten in der Behandlung	84,6 % Ja	7,3	2,8
4	Anzahl der Patient*innen ohne Glukokortikoide nach Erreichen einer Remission	100 % Ja	7,1	2,3
5	Interkollegialer und intersektoraler Austausch	92,3 % Ja	5,4	2,3
6	Interdisziplinäre Versorgung	92,3 % Ja	5,5	2,4
7	Rehabilitation bei Patient*innen mit eingeschränkter Teilhabe verordnen	92,3 % Ja	5,8	2,9
8	Verordnung von Heil- und Hilfsmitteln bei Patient*innen mit Einschränkung der Funktionsfähigkeit	92,3 % Ja	7,0	2,3
9	Selbstmanagement der Patient*innen	84,6 % Ja	4,6	2,7
9a	Selbstmanagement der Patient*innen: Strukturierte Patient*innenschulung bei jedem*r Patient*in	92,3 % Ja	6,8	2,9
9b	Selbstmanagement der Patient*innen: Jede*r Patient*in soll über den Zugang zur Selbsthilfeorganisation informiert werden	53,8 % Ja	3,4	2,9
10	Prävention sowie Diagnostik und Therapie einer begleitenden Osteoporose bei RA	84,6 % Ja	5,8	2,7
11	Regelmäßige Erhebung der Krankheitsaktivität	53,8 % Ja	4,7	3,0
12	Differenzialdiagnose der Schmerzen und Zugang zum Schmerzmanagement	76,9 % Ja	6,5	3,1
13	Notfallmanagement/kurzfristiger Zugang/schneller Termin	92,3 % Ja	7,9	2,4
14	Konsequente Therapieeskalation bei Nichterreichen einer Remission	100 % Ja	7,2	2,0
14a	Konsequente Therapieeskalation bei Nichterreichen einer Remission: medikamentöse Eskalation oder Wechsel	100 % Ja	6,9	2,0
14b	Konsequente Therapieeskalation bei Nichterreichen einer Remission: Durchführung von Punktionen/Injektionen	92,3 % Ja	6,8	2,3
15	Screening psychosozialer Folgeprobleme	100 % Ja	6,6	1,7
16	Jährlicher Review	69,2 % Ja	5,6	3,2
17	Kontrolle Impfstatus und Ergänzung der Impflücken	92,3 % Ja	6,8	2,4
18	Behandlungsplan zwischen Ärzt*in und Patient*in	69,2 % Ja	3,8	3,6
19	Angebote effizienter, digitaler Medien als Ergänzung zur ärztlichen Versorgung	61,5 % Ja	4,9	3,6

*QS* Qualitätsstandard, *SD* Standardabweichung

Bei der Diskussion spielten die Aspekte der Validität inklusive der Augenscheinvalidität, der Durchführbarkeit, der inhaltlichen Bedeutung des Schlüsselbereiches und der Wahrscheinlichkeit der Verwendung eine Rolle.

#### Phase 3: Priorisierung der Versorgungslücken

Die Priorisierung wurde in einem weiteren virtuellen Treffen im Dezember 2020 vorgestellt und intensiv diskutiert. Die AG entschied sich, insgesamt zu 8 Versorgungslücken korrespondierende QS zu formulieren. Die Entscheidung fußte auf einer balancierten Abwägung zwischen Versorgungslücken, die zu 100 % als relevant bewertet wurden und Versorgungslücken, die einen hohen Grad der Zustimmung erhielten.

#### Phase 4: Formulierung der Qualitätsstandards für RA

Die Ausformulierung der QS erfolgte im Rahmen eines virtuellen Meetings, an dem 13 Mitglieder der AG teilnahmen. Die finale Version der QS findet sich in den Tab. 3, 4, 5, 6, 7, 8, 9 und 10 und die ausführliche Besprechung der inhaltlichen Diskussion weiter unten im Text. An der Abstimmung nahmen am Ende des Treffens alle 11 noch anwesenden Mitglieder der Kommission teil.

**Table 3:** 

Domäne	Standard	Rationale	Qualitätsmessung, Kategorie Struktur	Qualitätsmessung, Kategorie Prozess, Zähler	Qualitätsmessung, Kategorie Prozess, Nenner
Frühzeitige Diagnose	Bei Patient*innen mit neu aufgetretenen Gelenkschmerzen und Verdacht auf RA wird die Diagnose innerhalb von 6 Wochen gesichert	Die Symptome der RA, insbesondere die frühen, zum Teil unspezifischen Symptome, werden von Nicht-Rheumatolog*innen oft nicht als Manifestation einer entzündlich-rheumatischen Erkrankung erkannt. Dadurch unterbleibt die Durchführung diagnostischer Maßnahmen, und es kommt zu einer erheblichen Verzögerung bei Diagnose und Therapieeinleitung. Radiologisch nachweisbare Gelenkdestruktionen und Funktionseinschränkungen entwickeln sich gerade zu Beginn der Erkrankung am stärksten. Das Therapieziel für Patient*innen mit RA ist die Remission (s. QS 2). Das Erreichen einer Remission ist umso wahrscheinlicher, je früher die Diagnose einer RA gestellt und eine Therapie eingeleitet wird. Die Anzahl aufgetretener Erosionen korreliert mit der zeitlichen Verzögerung bis zur Einleitung der ersten Therapie. Wenn der Verdacht auf RA besteht, geben die EULAR-Empfehlungen der frühen RA, die ACR-Klassifikationskriterien und die S3-Leitlinie für frühe RA der DGRh eine Orientierung für die Diagnosefindung. Die Patient*innen, die sich mit Symptomen einer RA ärztlich vorstellen, sollen zügig in die Rheumatologie überweisen werden. Da es eine Vielzahl an Differenzialdiagnosen abzuklären gibt, ist die Fachexpertise der Rheumatologie erforderlich. Frühsprechstunden, in denen Patient*innen mit noch unklaren Arthralgien frühzeitig auf das mögliche Vorliegen einer RA untersucht werden, können wesentlich zur rechtzeitigen Diagnosestellung beitragen	Vorhaltung von Maßnahmen und Strukturen, die eine frühe Diagnose ermöglichen. Dies schließt nicht nur eine ausreichende Anzahl von Rheumatolog*innen und entsprechende Kapazitäten in Frühsprechstunden ein, sondern auch Maßnahmen zur Fortbildung der primärärztlichen Versorgungsebene und anderer Gesundheitsdienstleister, z. B. durch rheumatologische Zentren. Dazu zählen aber auch Informationsangebote für die Bevölkerung – auch durch die Deutsche Rheumaliga –, um die Sensibilisierung für Anzeichen und Frühsymptome einer RA zu erhöhen und zu fördern, damit Patient*innen mit Verdacht auf RA innerhalb von 6 Wochen diagnostiziert werden können	Alle neudiagnostizierten RA-Patient*innen, bei denen die Diagnose innerhalb von 6 Wochen nach Symptombeginn fachärztlich gestellt wurde	Alle RA-Patient*innen, bei denen im letzten Jahr eine RA fachärztlich neu diagnostiziert wurde

**Table 4:** 

Domäne	Standard	Rationale	Qualitätsmessung, Kategorie Struktur	Qualitätsmessung, Kategorie Prozess, Zähler	Qualitätsmessung, Kategorie Prozess, Nenner
Ziel Remission	Das Ziel der Behandlung von Patient*innen mit RA ist die Remission	Eine erhöhte Krankheitsaktivität bei RA ist mit erhöhter Mortalität, zunehmenden Gelenkdestruktionen und Funktionseinschränkung sowie entsprechenden sozioökonomischen Konsequenzen assoziiert. Mit dem raschen Erreichen einer Remission werden nicht nur krankheitsspezifische Komplikationen vermieden, sondern es wird auch die Wahrscheinlichkeit erhöht, dass die Remission über einen längeren Zeitraum anhält. Die S3-Leitlinie zur Diagnose einer frühen RA stellt fest, dass eine Remission nach 3 (spätestens 6) Monaten erreicht werden soll. Je früher die Remission erreicht wird, desto wahrscheinlicher hält diese auch länger an – und dies z. T. sogar mit reduzierter Dosis verordneter Medikamente. Die Krankheitsaktivität von Patient*innen mit RA sollte mit validierten Scores bewertet werden. Entsprechend der S3-Leitlinie zur Diagnose einer frühen RA, der S2e-Leitlinie zur Therapie einer RA der DGRh sowie den EULAR-Empfehlungen zum Management der frühen RA wird empfohlen, den „simplified disease activity score“ (SDAI), den „clinical disease activity index“ (CDAI) oder den „disease activity score“ (DAS28) zu verwenden. Demnach ist Krankheitsremission als SDAI < 3,3, CDAI < 2,8 oder – weniger spezifisch – DAS28 < 2,6 definiert. Das Ziel ist grundsätzlich nicht nur das Erreichen, sondern auch das Aufrechterhalten der erreichten Remission. Bei Nicht-Erreichen einer Remission, sollen alle Möglichkeiten einer intersektoralen Versorgung ausgeschöpft werden und weitere medikamentöse sowie nichtmedikamentöse Therapieverfahren zur Anwendung kommen. Dabei ist die konsequente Intensivierung der Therapie bei Nichterreichen einer Remission ein wesentlicher Baustein des Therapiekonzeptes (s. QS 4)	Vorhaltung von Maßnahmen und Strukturen, einschließlich einer ausreichenden Anzahl von Rheumatolog*innen, mit uneingeschränktem Zugang (z. B. keine Fallzahldeckelung) sowie Gewährleistung einer intersektoralen Therapie, um sicherzustellen, dass Patient*innen mit RA regelmäßig mit validierten Messinstrumenten evaluiert werden und die Therapie gemäß der S3-Leitlinie sowie der S2e-Leitlinie mit dem Ziel der Remission angepasst werden kann	RA-Patient*innen in Remission	Alle Patient*innen mit RA

**Table 5:** 

Domäne	Standard	Rationale	Qualitätsmessung, Kategorie Struktur	Qualitätsmessung, Kategorie Prozess, Zähler	Qualitätsmessung, Kategorie Prozess, Nenner
Glukokortikoidfreiheit	Bei Patient*innen mit RA in Remission ist Glukokortikoidfreiheit das Ziel	Eine dauerhafte Glukokortikoidtherapie führt zu einer Vielzahl unerwünschter Wirkungen. Hierzu zählen unter anderem kardiovaskuläre Ereignisse, erhöhte Infektanfälligkeit, mit Frakturen einhergehende Osteoporose, gastrointestinale Komplikationen und auch selten eine sekundäre Nebennierenrindeninsuffizienz. Die glukokortikoidfreie Remission ist daher das primäre Ziel der Therapie. Gemäß der S3-Leitlinie zur Diagnose einer frühen RA, der S2e-Leitlinie zur Therapie der RA sowie den EULAR-Empfehlungen zum Management einer (frühen) RA der DGRh wird bis zum Erreichen der Wirkung einer csDMARD-Therapie die Krankheitsaktivität mit einer Glukokortikoidtherapie unterdrückt. Die Glukokortikoiddosis soll dann aber innerhalb von 8 Wochen in den Low-Dose-Bereich (≤ 7,5 mg/Tag Prednisolonäquivalent) reduziert und nach 3 bis 6 Monaten nach Möglichkeit beendet werden. Bei Patient*innen, bei denen das vollständige Absetzen der Glukokortikoide nicht erreichbar ist, wird eine Dosis von maximal 5 mg Prednisolonäquivalent/Tag als oberste akzeptable Grenze definiert	Vorhaltung von Maßnahmen und Strukturen, einschließlich des Zugangs zu allen medikamentösen Therapiemöglichkeiten, um die Chance auf eine glukokortikoidfreie Remission zu maximieren	RA-Patient*innen in Remission ohne Glukokortikoide	Alle RA-Patient*innen in Remission

**Table 6:** 

Domäne	Standard	Rationale	Qualitätsmessung, Kategorie Struktur	Qualitätsmessung, Kategorie Prozess, Zähler	Qualitätsmessung, Kategorie Prozess, Nenner
Konsequente Therapieanpassung	Bei Nichterreichen der Remission gilt es, die Ursachen differenzialdiagnostisch aufzuarbeiten und die Therapie entsprechend zu adaptieren	Eine nicht unbedeutende Zahl von Patient*innen gilt als therapierefraktär oder schwer behandelbar, trotz korrekter Diagnosestellung und regelmäßiger Evaluation der Krankheitsaktivität. Für die Versorgung dieser Patient*innen bieten die aktuellen EULAR-Empfehlungen für die „Difficult-to-treat“-RA sowie die S2e-Leitlinie zur Therapie einer RA der DRGh evidenzbasierte Informationen. Dabei gilt es, differenzialdiagnostische Überlegungen hinsichtlich der Ursache des Nichterreichens oder des sekundären Verlustes der Remission anzustellen. Da die Unterscheidung zwischen bestehender entzündlich bedingter Krankheitsaktivität und anderen Ursachen wie sekundären Schmerzerkrankungen und Arthrose zum Teil schwierig zu differenzieren ist, bedarf es der Fachexpertise von Rheumatolog*innen. Bei Nichterreichen oder einem sekundären Verlust einer Remission, sollen alle Möglichkeiten einer intersektoralen Versorgung ausgeschöpft werden und medikamentöse sowie nichtmedikamentöse Therapieverfahren, wie z. B. Kälte- oder Wärmetherapie, Physiotherapie und/oder Ergotherapie zur Anwendung kommen. Die medikamentöse Therapie muss überprüft und ggf. umgestellt werden. Darüber hinaus können interventionelle Verfahren wie eine intraartikuläre Glukokortikoidinjektion erforderlich sein. Die Entwicklung von Komorbiditäten ist bei der Therapieentscheidung mit zu berücksichtigen	Vorhaltung von Maßnahmen und Strukturen einschließlich einer ausreichenden Anzahl von Rheumatolog*innen (Facharztstandard), um bei Nichterreichen einer Remission gezielte differenzialdiagnostische Überlegungen anzustellen und einen uneingeschränkten Zugang zu allen Therapiemöglichkeiten zu ermöglichen, um notwendige Therapieadaptationen durchführen zu können	RA-Patient*innen, bei denen eine Therapieadaptation bei Nichterreichen einer Remission vorgenommen wurde	Alle RA-Patient*innen, die nicht in Remission sind

**Table 7:** 

Domäne	Standard	Rationale	Qualitätsmessung, Kategorie Struktur	Qualitätsmessung, Kategorie Prozess, Zähler	Qualitätsmessung, Kategorie Prozess, Nenner
Konsequente Therapie der eingeschränkten Funktionsfähigkeit	RA-Patient*innen mit Einschränkung der Funktionsfähigkeit werden therapeutische Maßnahmen zur Wiederherstellung der Funktionsfähigkeit angeboten	Im Laufe der Erkrankung kann es infolge unzureichend kontrollierter Krankheitsaktivität zu Gelenkdestruktionen und Funktionseinschränkungen kommen. Es ist Aufgabe des/der Rheumatolog*in, die Funktionseinschränkungen mit validierten Messinstrumenten (FFbH und/oder HAQ sowie der Neutral-Null-Methode) festzustellen und zu dokumentieren. Die Schwellenwerte für eine Funktionseinschränkung liegen laut Expertenkonsensus und vorausgegangenen FFbH-Itemanalysen im Datensatz der Kerndokumentation bei einem FFbH ≤ 70 % und/oder einem HAQ von ≤ 0,5. Im Rahmen dessen muss der/die Rheumatolog*in unter Berücksichtigung bestehender Komorbiditäten einschätzen, ob die festgestellte Funktionseinschränkung Folge der RA ist. Dabei kommt der Unterscheidung zwischen Krankheitsaktivität und Strukturschaden hinsichtlich der Abschätzung des Therapieerfolges eine wesentliche Bedeutung zu. Bei bestimmten Patient*innen (z. B. bei Multimorbidität) kann die klinische Bewertung auch ohne entsprechende Patient*innenfragebögen ausreichen. Wurde ein Funktionsdefizit dokumentiert, ist es Aufgabe der/des Rheumatolog*in, entsprechende Verordnungen für Rehabilitationsmaßnahmen, Heil- und Hilfsmittel sowie ggf. eine akutstationäre Einweisung zur Komplextherapie zu veranlassen. Darüber hinaus ist es Aufgabe des/der Rheumatolog*in, die Durchführung und den Erfolg der verordneten Maßnahmen zu kontrollieren und zu dokumentieren. Hierzu ist eine enge Interaktion zwischen behandelnden Physio- und Ergotherapeut*innen und Rheumatolog*innen erforderlich	Vorhaltung von Maßnahmen und Strukturen, einschließlich einer ausreichenden Anzahl von rheumatologischem Fachpersonal (Rheumatolog*innen und rheumatologischer Fachassistenz [RFA]) sowie von Physio- und Ergotherapeut*innen mit ausreichenden Kenntnissen hinsichtlich rheumatologischer Funktionseinschränkungen, um das Auftreten von Funktionseinschränkungen regelmäßig zu erfassen, zu dokumentieren und zu behandeln. Neben der personellen Ressource muss auch eine ausreichend hohe Kapazität an rheumatologisch zugewiesenen Betten für die akutstationäre sowie rehabilitative Versorgung vorhanden sein Gemäß der aktuellen Heilmittel-Richtlinie darf es bei entsprechender Notwendigkeit der Therapien keine Budgetierung geben	Alle RA-Patient*innen mit Funktionseinschränkung, denen Rehabilitationsmaßnahmen, Heil- und Hilfsmittel und/oder akutstationäre Komplextherapie angeboten wurden	Alle RA-Patient*innen mit Funktionseinschränkungen

**Table 8:** 

Domäne	Standard	Rationale	Qualitätsmessung, Kategorie Struktur	Qualitätsmessung, Kategorie Prozess, Zähler	Qualitätsmessung, Kategorie Prozess, Nenner
Screening psychosozialer Folgeprobleme	Bei jedem/jeder Patient*in mit RA werden psychosoziale Folgeprobleme erkannt, dokumentiert und Lösungen angestrebt	Obwohl sie häufig vorkommen, werden psychische Begleiterkrankungen der RA wie Depression oder Angststörungen oft nur unzureichend diagnostiziert bzw. bei einer gewissen Tabuisierung dieses Themas nicht aktiv angesprochen und selten therapiert. Dabei können depressive Symptome eine Remission der Erkrankung bereits im frühen Krankheitsstadium ungünstig beeinflussen. Des Weiteren werden Fragestellungen zur Sexualität und Familienplanung der Patient*innen mit RA häufig nicht aktiv angesprochen. Ebenso sind bzw. werden viele Patient*innen nur unzureichend über Möglichkeiten der Arbeitsplatzumgestaltung bzw. der Änderung der Erwerbssituation, einer Minderung der Erwerbsfähigkeit, die Beantragung eines Schwerbehindertenausweises sowie eine mögliche Pflegegradbeantragung informiert. Aufgabe des/der Rheumatolog*in ist es, psychosoziale Folgeprobleme und Fragestellungen (Depression, Angst, Fatigue, Erwerbsfähigkeit, Arbeitsplatz, Kognition, geriatrische Fragestellungen, Familienplanung, Pflegegrad) klinisch inklusive einer Sozial- und Berufsanamnese einzuschätzen und, wenn möglich, mit validierten Fragebögen standardisiert zu erfassen und die Patient*innen, ggf. in Kooperation mit weiteren Fachärzt*innen (z. B. Rehabilitationsmediziner*innen und/oder Arbeits-und Sozialmediziner*innen und/oder Psycholog*innen sowie Psychiater*innen), in der Verbesserung der Teilhabe und Aktivität zu unterstützen	Vorhaltung von Maßnahmen und Strukturen, einschließlich einer fundierten Lehre der Sozialmedizin im Medizinstudium, einer ausreichenden Anzahl an sozialmedizinisch geschulten Rheumatolog*innen sowie Rehabilitationsmediziner*innen, Arbeits-und Sozialmediziner*innen, Psycholog*innen und Psychiater*innen mit Kenntnissen hinsichtlich RA, um eine interdisziplinäre Versorgung psychosozialer Folgeprobleme im Hinblick auf die RA sicherzustellen. Ebenso sollten die Patient*innen im Rahmen von Patient*innenschulungen zum Empowerment befähigt werden	Anzahl der Patient*innen mit RA, bei denen psychosoziale Folgeprobleme angesprochen und Lösungsansätze initiiert wurden	Alle Patient*innen mit RA

**Table 9:** 

Domäne	Standard	Rationale	Qualitätsmessung, Kategorie Struktur	Qualitätsmessung, Kategorie Prozess, Zähler	Qualitätsmessung, Kategorie Prozess, Nenner
Notfall- und Akutmanagement	Patient*innen mit RA und akuten medizinischen Problemen oder Fragestellungen hinsichtlich ihrer rheumatologischen Versorgung wird eine notfallmäßige Vorstellung bzw. schnelle Kontaktaufnahme zur Klärung der Problematik bei Rheumatolog*innen ermöglicht	Patient*innen mit RA haben im Vergleich zur Normalbevölkerung eine erhöhte Mortalität. Diese ist der entzündlichen Grunderkrankung, den Komorbiditäten und der erhöhten Infektanfälligkeit, zum Teil infolge der Medikation, geschuldet. RA-Patient*innen sind deshalb gefährdet, akute Gesundheitsprobleme wie Krankheitsschübe oder therapiebedingte Komplikationen zu erleiden, die einer zeitnahen ärztlichen Versorgung bedürfen. Hierbei sind rheumatologische Notfälle von anderen aus Patient*innensicht dringenden Versorgungsfragen zu differenzieren. Während gewährleistet sein muss, dass Patient*innen sich bei medizinischen Notfällen unverzüglich bei ihrem/ihrer behandelnden Rheumatolog*in vorstellen können, reicht zum Teil auch eine Kontaktaufnahmemöglichkeit bis zum nächsten Werktag – auch unter Ausschöpfung telefonischer bzw. digitaler Optionen – bei dringenden Versorgungsfragen aus. Patient*innen mit RA müssen die Strukturen der Kontaktaufnahme bekannt gemacht werden. Um eine optimale vertragsärztliche bzw. ambulante spezialfachärztliche (ASV) und/oder stationäre Versorgung anbieten zu können, ist eine enge Kooperation zwischen Vertragsärzt*innen und Kliniken erforderlich, sodass bei entsprechendem Bedarf eine stationäre Aufnahme in einer rheumatologischen Fachklinik umgehend realisiert werden kann	Vorhaltung von Maßnahmen und Strukturen, einschließlich eines intersektoralen Notfallversorgungskonzeptes, in dem ein enger Verbund zwischen jedem/jeder niedergelassenen Rheumatolog*in mit der nächstgelegenen stationären rheumatologischen Fachklinik besteht, um die notfallmäßige Versorgung rheumatologischer Notfälle bzw. das Akutmanagement von Patient*innen mit RA zu gewährleisten	Rheumatologische Notfälle: Anzahl der Patient*innen mit RA und akuten gesundheitlichen Problemen, die unverzüglich fachrheumatologisch gesehen wurden Versorgungsfragen: Anzahl der Patient*innen mit RA und Versorgungsfragen, die bis zum nächsten Werktag kontaktiert wurden	Anzahl der RA-Patient*innen mit (sub)akuten gesundheitlichen Problemen im Rahmen der rheumatologischen Versorgung

**Table 10:** 

Domäne	Standard	Rationale	Qualitätsmessung, Kategorie Struktur	Qualitätsmessung, Kategorie Prozess, Zähler	Qualitätsmessung, Kategorie Prozess, Nenner
Komorbiditäten – Erfassung/Management	Bei Patient*innen mit RA werden 1‑mal jährlich Komorbiditäten erfasst und bei entsprechender Notwendigkeit therapeutische Konsequenzen eingeleitet	Die häufigste Todesursache bei Patient*innen mit RA sind kardiovaskuläre Erkrankungen. Das Risiko für kardiovaskuläre Komorbidität ist insgesamt 1,5- bis 2‑fach erhöht. Auch andere Komorbiditäten bzw. krankheitsbedingte Komplikationen stellen ein Risiko für Patient*innen mit RA dar: z. B. Osteoporose mit der Gefahr von Frakturen oder infektiöse Komplikationen. Daher beinhaltet die internistisch-rheumatologische Betreuung der RA-Patient*innen, dass Komorbiditäten erkannt, dokumentiert und eine Therapieeinleitung in Kooperation mit der*dem Hausarzt*ärztin und anderen Spezialist*innen veranlasst wird. Dazu gehören eine mindestens jährliche Blutdruckmessung und Erhebung der Risikofaktoren für Osteoporose – einschließlich der Sturz- und Frakturanamnese für das letzte Jahr. Gemäß den EULAR-Empfehlungen für das Management des kardiovaskulären Risikos sollte dieses alle 5 Jahre oder nach Änderung der antirheumatischen Therapie erfasst werden. Hierbei sollten kardiovaskuläre Risikofaktoren (Raucherstatus, Body-Mass-Index, Anamnese hinsichtlich eines Bluthochdrucks/einer Hypercholesterinämie/einer chronischen Niereninsuffizienz), die Anamnese hinsichtlich zurückliegender Schlaganfälle, Myokardinfarkte mit ggf. erfolgten Interventionen, Angina pectoris, TIA, Herzinsuffizienz sowie pAVK und aktuelle Therapien bezüglich kardiovaskulärer Erkrankungen (antihypertensive Therapien, Thrombozytenaggregationshemmung, antidiabetische Therapien inklusive Insulintherapien, lipidsenkende Therapien und Antikoagulanzien) abgefragt und dokumentiert werden. Ferner sollten Lipide im Blut immer in Remission oder stabiler Krankheitsaktivität bestimmt werden, weil die Messwerte bei entzündlicher Aktivität oft falsch niedrig sind. Weiterhin gehört der Hinweis auf die Impfempfehlungen der ständigen Impfkommission (STIKO), die Notwendigkeit regelmäßiger Vorsorgeuntersuchungen sowie eine Überprüfung der aktuellen Medikamentenliste zur jährlichen Überprüfung. Die Durchführung bzw. weitere Organisation solcher Maßnahmen durch andere Fachärzt*innen kann an den*die Hausarzt*ärztin delegiert werden. Diesbezüglich ist es sinnvoll, den Patient*innen im Rahmen des jährlichen Gespräches eine Information über durchzuführende Impfungen oder Screeninguntersuchungen für den/die Hausarzt*ärztin mitzugeben, in welchem über empfohlene Impfungen oder Screeninguntersuchungen informiert wird. Neben den STIKO-Empfehlungen sind hier die EULAR-Empfehlungen für Impfungen bei erwachsenen Patienten mit entzündlich-rheumatischen Erkrankungen maßgebend. Dessen ungeachtet, sollen Patient*innen im Rahmen von Patient*innenschulungen gemäß den EULAR-Empfehlungen für Patient*innenschulung regelmäßig zu informiertem selbstverantwortlichem Krankheitsmanagement befähigt werden. Die Entscheidungen sollten in einer partizipativen Entscheidungsfindung zwischen Ärzt*innen und Patient*innen gefällt werden („Shared-decision-Prozess“)	Vorhaltung von Maßnahmen und Strukturen einschließlich der Erstellung von standardisierten Informationsbögen für Hausärzt*innen, um eine gute interdisziplinäre Zusammenarbeit bei der Behandlung von Komorbiditäten der RA-Patient*innen sowie empfohlenen Impfungen zu gewährleisten	Anzahl der Patient*innen mit RA und einer Diagnosedauer ≥ 1 Jahr, bei denen Komorbiditäten innerhalb der letzten 12 Monate erfasst und daraus entsprechende therapeutische Konsequenzen gezogen wurden	Alle Patient*innen mit RA und einer Diagnosedauer ≥ 1 Jahr

#### Phase 5: Konsentierung der Qualitätsstandards

An der Abstimmung der QS nahmen insgesamt 13 AG-Mitglieder teil. Der niedrigste Grad der Zustimmung fand sich bei QS 6 (Screening psychosozialer Folgeprobleme) mit 8,7 ± 1,9 und der höchste Grad der Zustimmung bei QS 4 (Konsequente Therapieanpassung) mit 9,9 ± 0,3. Im Durchschnitt lag der Grad der Zustimmung aller QS bei 9,3 ± 0,7. Die Abstimmungsergebnisse der QS sind in Tab. 11 zu finden.

**Table 11:** 

Qualitätsstandard	Thema	Zustimmung ja/nein	Grad der Zustimmung (NRS 0–10)
QS 1	Frühzeitige Diagnose	13/0	9 (2,2)
QS 2	Ziel Remission	13/0	9,8 (0,4)
QS 3	Glukokortikoidfreiheit	13/0	9,3 (1,0)
QS 4	Konsequente Therapieanpassung	13/0	9,9 (0,3)
QS 5	Konsequente Therapie der eingeschränkten Funktionsfähigkeit	13/0	9,4 (1,1)
QS 6	Screening psychosozialer Folgeprobleme	13/0	8,7 (1,9)
QS 7	Notfall- und Akutmanagement	13/0	9,4 (1,0)
QS 8	Komorbiditäten – Erfassung/Management	13/0	9,2 (1,2)

### Qualitätsstandards für die Versorgung von Patient*innen mit rheumatoider Arthritis

#### QS 1: Frühzeitige Diagnose (Tab. 3)

Bei der Formulierung dieses QS wurde das Hauptaugenmerk auf eine zeitnahe Diagnosestellung gelegt, da sich alle AG-Mitglieder darin einig waren, dass jede Woche zählt, um das Ziel der Remission so früh wie möglich zu erreichen. Grundsätzlich wurde angemerkt, dass es bereits initial zu Verzögerungen kommen kann, da der Beginn von Diagnostik und Therapie vom Zeitpunkt der Erstvorstellung des*r Patient*in bei einem*r Ärzt*in, in der Regel bei einem*r internistisch-rheumatologischen Facharzt*ärztin abhängig ist. Diese Vorstellung hängt unter anderem erheblich vom Leidensdruck des*r Patient*in bzw. seinem*ihrem Interesse ab, die Beschwerden zügig abzuklären. Insofern darf es in den weiteren Schritten (hausärztliche Überweisung, rheumatologische Erstvorstellung) keine weiteren Verzögerungen geben. Die Expertise eines*r internistisch-rheumatologischen Facharzt*ärztin ist sowohl für die Diagnosestellung als auch für die Therapieeinleitung in den meisten Fällen erforderlich, um unnötige und die Patient*innen potenziell schädigende Folgen zu vermeiden. Deshalb werden in den meisten rheumatologischen Einrichtungen Frühsprechstunden angeboten. In manchen Fällen ist auch die Einweisung in ein rheumatologisches Akutkrankenhaus erforderlich.

Nach intensiven inhaltlichen Diskussionen stimmten alle 11 anwesenden Mitglieder für diesen QS.

#### QS 2: Ziel Remission (Tab. 4)

Zum einen wurde hier diskutiert und festgestellt, dass eine komplette Remission nicht bei allen RA-Patient*innen zu erreichen ist. Dieser Ansicht waren zwar fast alle AG-Mitglieder, jedoch wurde auch kritisch angemerkt, dass eine Teilremission kein Qualitätsmerkmal darstellen kann. Insofern wurde entschieden, dass zwar das primäre Ziel die komplette Remission ist, jedoch davon auszugehen ist, dass diese Qualität nicht bei allen RA-Patient*innen zu erreichen ist. In der internationalen Literatur wird dieses durchaus bekannte Problem meist so gelöst, dass von einer möglichen Akzeptanz niedriger Krankheitsaktivität gesprochen wird [35, 36].

Zum anderen wurden die zur Verwendung kommenden validierten Scores zur Bestimmung der Krankheitsaktivität diskutiert. Hier wurde kritisch angemerkt, dass der DAS28 durch die darin nicht enthaltene Beurteilung der Vorfüße den Gelenkstatus nur unzureichend abbildet [37]. Zudem wurde kritisch diskutiert, dass das Nichterreichen einer Remission möglicherweise durch eine hohes Patientenglobalurteil verursacht sein könnte, wobei dadurch dann die Selbstbeurteilung im Missverhältnis zur Gelenkuntersuchung steht [38].

Die aktuellen S2e- sowie S3-LL der DGRh [2, 3] empfehlen zur Bestimmung der Krankheitsaktivität den CDAI, SDAI und/oder den DAS28. Basierend auf diesen Empfehlungen wurde entschieden, dass alle 3 Scores zur Bestimmung der Krankheitsaktivität verwendet werden können.

Nach intensiven inhaltlichen Diskussionen stimmten alle 11 anwesenden Mitglieder für diesen QS.

#### QS 3: Glukokortikoidfreiheit (Tab. 5)

Generell bestand Einigkeit, dass eine möglichst niedrige Glukokortikoiddosis bei RA-Patient*innen anzustreben ist und dass das Ziel bei Patient*innen in Remission die Glukokortikoidfreiheit ist. Über die Höhe der Glukokortikoiddosis gab es allerdings eine intensive Diskussion zwischen den AG-Mitgliedern, da die nationalen und internationalen Leitlinien Dosierungen zwischen < 5 und < 7,5 mg Prednisolonäquivalent angeben [2, 3, 39]. Die AG-Mitglieder einigten sich auf die Formulierung, einen zeitlich begrenzten Dosisbereich von ≤ 7,5 mg Prednisolonäquivalent/Tag zu akzeptieren, und präzisierten, dass bei Patient*innen, bei denen das vollständige Absetzen der Glukokortikoide nicht erreichbar ist, eine Dosis von maximal 5 mg Prednisolonäquivalent/Tag als oberste akzeptable Grenze definiert wird.

Nach intensiven inhaltlichen Diskussionen stimmten alle 11 anwesenden Mitglieder für diesen QS.

#### QS 4: Konsequente Therapieanpassung (Tab. 6)

Das wichtige Therapieziel Remission wird aus verschiedenen Gründen relativ häufig nicht erreicht ([36, 40, 41], s. auch QS 2). Das Hauptaugenmerk in der Diskussion bei diesem QS lag in der möglicherweise fehlenden, aber zum Teil durchaus notwendigen Ursachenforschung für die nicht erreichte Remission durch den*die Rheumatolog*in. Dabei wurde betont, dass das frühzeitige Erkennen von begleitenden schmerzbegünstigenden Erkrankungen wie Arthrose und Fibromyalgie differenzialdiagnostisch von großer Bedeutung ist. Daher wurde in diesem QS nicht nur die konsequente Therapieanpassung bei nicht erreichter Remission, sondern auch „[…] die differenzialdiagnostische Aufarbeitung der Ursache(n) […]“ als klares Ziel für diesen QS formuliert.

Nach intensiven inhaltlichen Diskussionen stimmten alle 11 anwesenden Mitglieder für diesen QS.

#### QS 5: Konsequente Therapie der eingeschränkten Funktionsfähigkeit (Tab. 7)

Bei diesem QS wurden verschiedene Aspekte diskutiert. Grundsätzlich bestehen bei Patient*innen mit RA häufig Komorbiditäten, die zu entsprechenden Funktionseinschränkungen führen können. Ein wichtiges Ziel des*r Rheumatolog*in ist daher die Differenzierung solcher Ursachen von Funktionseinschränkungen von der durch die RA bedingten eingeschränkten Funktionsfähigkeit. Hierbei spielt auch die Unterscheidung zwischen einer akut eingeschränkten Funktionsfähigkeit durch aktive Entzündung und die durch einen bereits eingetretenen strukturellen Schaden, wie z. B. durch sekundäre Arthrose, eine Rolle.

Darüber hinaus wurde kritisch angemerkt, dass es keine validierten Scores gibt, die einen Schwellenwert für eingeschränkte Funktionsfähigkeit festlegen. Die nun in dem QS verwendeten Schwellenwerte bezüglich eingeschränkter Funktionsfähigkeit basieren auf Expert*innenmeinung sowie auf Auswertungen der Kerndokumentation [42]. In diesem Zusammenhang wird aber auf die grundsätzliche Limitation solcher Schwellenwerte verwiesen, da diese u. a. nicht das Alter, den BMI, (Fehlen) körperlicher Aktivität und den Lebensstil und den damit einhergehenden Funktionsverlust berücksichtigen [43].

Außerdem gibt es erhebliche individuelle Unterschiede bei Patient*innen mit RA in der Krankheitsverarbeitung, was z. B. das Coping mit der Krankheit mit einschließt und dass zum anderen auch die Umgebungssituation eine nicht geringe Rolle spielen kann – hierbei geht es um Verwandte, Freunde und die möglicherweise nicht behindertengerechte Wohnsituation oder auch die Verhältnisse am Arbeitsplatz.

Nach intensiven inhaltlichen Diskussionen stimmten alle 11 anwesenden Mitglieder für diesen QS.

#### QS 6: Screening psychosozialer Folgeprobleme (Tab. 8)

Psychosoziale Folgeprobleme können für Patient*innen und Rheumatolog*innen mit RA in der Kommunikation ein schwierig anzusprechendes Thema sein, daher war den meisten AG-Mitgliedern ein gesonderter QS besonders wichtig. Hierbei stand nicht nur das Erkennen von entsprechenden Problemen (s. auch QS 5) im Vordergrund, sondern auch, ggf. in Kooperation mit anderen Fachabteilungen, das Einleiten von Maßnahmen, um Probleme ggf. zu beheben oder gegebene Einschränkungen zu verbessern. In der Diskussion dieses QS wurde daher ein großes Augenmerk auf die interdisziplinäre Zusammenarbeit bei der Lösung psychosozialer Probleme gelegt. Die psychische Komponente spielt nicht nur in der Lebensqualität der Patient*innen eine Rolle, sondern beeinflusst auch erheblich das Erreichen einer Remission. Verlaufsbeobachtungen zeigen, dass RA-Patient*innen mit depressiven Symptomen im weiteren Verlauf deutlich seltener und langsamer eine Remission erreichen als RA-Patient*innen ohne ein solches Problem [44].

Nach intensiven inhaltlichen Diskussionen stimmten alle 11 anwesenden Mitglieder für diesen QS.

#### QS 7: Notfall- und Akutmanagement (Tab. 9)

Ein strittiges Thema war die Definition eines rheumatologischen „Notfalls“. Hier bestand Einigkeit, dass es eine möglichst klare Differenzierung zwischen einem (objektiven) medizinischen Notfall und einem (subjektiven) individuellen akuten Versorgungsbedarf geben sollte. Da die Abgrenzung im Einzelfall jedoch schwierig sein kann, einigten sich die Mitglieder nach langer Diskussion dann auf diese hier gegebene Formulierung für das „Notfall- und Akutmanagement“. Bei der Umsetzung sind nach Meinung der Kommissionsmitglieder dann v. a. auch Aspekte der Machbarkeit von Bedeutung.

Nach intensiven inhaltlichen Diskussionen stimmten alle 11 anwesenden Mitglieder für diesen QS.

#### QS 8: Komorbiditätenerfassung/Management (Tab. 10)

Eine Haupttodesursache von RA-Patient*innen sind nach wie vor kardiovaskuläre Ereignisse [45]. Daher wurde von einigen AG-Mitgliedern befürwortet, dass das Screening auf kardiovaskuläre Risikofaktoren – wie von der EULAR empfohlen [46] – einen eigenen QS darstellen sollte. Dementgegen gaben jedoch andere AG-Mitglieder zu bedenken, dass in der aktuellen Versorgungslandschaft in Deutschland der*die Rheumatolog*in keineswegs allein oder hauptamtlich für das Screening auf Komorbiditäten verantwortlich ist und daher hierfür nicht 2 separate QS gebildet werden müssen. Letztlich wurde sich darauf geeinigt, einen ausführlichen QS zu entwickeln, in dem alle relevanten Komorbiditäten gleichermaßen gewürdigt werden und auf die Notwendigkeit einer aktiven Teilnahme des*der Hausarzt*ärztin sowie weiterer Fachexpert*innen hingewiesen bzw. diese auch eingefordert wird.

Nach intensiven inhaltlichen Diskussionen stimmten alle 11 anwesenden Mitglieder für diesen QS.

## Diskussion

Die hier erstmals veröffentlichten QS der DGRh in Kooperation mit VRA und BDRh stellen für die rheumatologische Versorgung in Deutschland einen Meilenstein dar. Die Expertengruppe hat 8 QS für wichtige Bereiche der Versorgung von RA-Patient*innen definiert. Zum ersten Mal gibt es damit auch eine Grundlage und Vorgaben für Qualitätsmessungen, wobei nicht nur die Struktur- und Prozessqualität, sondern auch die Ergebnisqualität eine Rolle spielen sollte (s. unten).

Die hier vorgestellten QS basieren auf den Vorarbeiten und verschiedenen anderen Initiativen der DGRh [47–50]. Hinsichtlich der Strukturqualität gibt es für internistisch-rheumatologische Akutkrankenhäuser bereits seit 10 Jahren eine klare Festlegung [24] und seit einigen Jahren auch ein vom AQUA-Institut verliehenes Gütesiegel [51], was unter anderem ein erfolgreiches Benchmarking von mehreren im VRA organisierten Kliniken beinhaltet [52]. Die hiermit verbundenen Projekte OBRA („outcome benchmarking“ in der rheumatologischen Akutversorgung) [23] und KOBRA [22, 51] wurden initial vom Bundesministerium für Gesundheit gefördert.

In den hier entwickelten QS geht es v. a. um Prozess-, aber auch um Ergebnisqualität. Da stehen die rechtzeitige Diagnosestellung und Therapieeinleitung im Fokus, die natürlich auch von vielen externen Faktoren wie einer rechtzeitigen Überweisung abhängen, aber auch von internen Prozess- und Strukturmerkmalen wie einem funktionierenden Angebot an Frühsprechstunden und der Verfügbarkeit einer ausreichenden Zahl an internistischen Rheumatolog*innen [49]. Vor allem bestehen Probleme in Form einer begrenzten Anzahl an Weiterbildungsstellen, der Konzentration auf besonders attraktive Regionen und Ballungsräume wie etwa Berlin, München oder Hamburg und einer bis jetzt fehlenden staatlichen Förderung und Planung von Weiterbildungsstellen. Nichtsdestoweniger geben die vorhandenen internistisch-rheumatologischen Einrichtungen ihr Bestes, um die beträchtliche Zahl von Patient*innen mit entzündlich rheumatischen Erkrankungen adäquat zu versorgen. Als das Fach in der Medizin, in welchem bei den betroffenen Patient*innen so gut wie alle Organsysteme betroffen sein können – bei der RA ist es v. a. die Lunge [45] –, ist die internistische Rheumatologie genuin interdisziplinär aufgestellt [53], was sich entsprechend auch in aktuellen Versorgungsstrukturen wie der ambulanten spezialfachärztlichen Versorgung (ASV) konzeptionell niederschlägt [54].

Das Therapieziel Remission ist in der internationalen Rheumatologie unstrittig. Im Wesentlichen umfasst es die konsequente regelmäßig überprüfte weitgehende Abwesenheit von Krankheitsaktivität. Die gängigen Definitionen für Remissionen beinhalten nicht die vollständige oder weitestgehende Abwesenheit von Krankheitsaktivität wie die Boolean-Kriterien [55]. Dass der Prozentsatz von RA-Patient*innen, bei denen Remission erreicht werden kann, ein gutes mögliches Kriterium darstellt, zeigt z. B. auch die CAPEA-Studie an, in der 40 % der eingeschlossenen RA-Patient*innen eine Remission erreichten [56]. Durch frühzeitige Diagnose und Therapie und stringente Befolgung des T2T-Prinzips wie im rheinland-pfälzischen Rheumanetzwerk ADAPTHERA [57] lassen sich deutlich höhere Werte erzielen.

Glukokortikoide sind klinisch gut und schnell wirksam, haben in hohen Dosierungen über lange Zeiträume aber gut bekannte Nebenwirkungen wie Osteoporose, Diabetes und Infektionen [39, 58]. Deshalb empfiehlt die EULAR eine wesentliche Reduktion bzw. das Absetzen von Glukokortikoiden innerhalb von 6 Monaten [39]. Dies wird aber bekanntermaßen von vielen Patient*innen nicht erreicht [56]. Auf der anderen Seite sind auch geringe Dosierungen < 5 mg Prednisolonäquivalent/Tag klinisch und sogar hinsichtlich der Röntgenprogression wirksam [59]. Deshalb geht es dabei v. a. um den Versuch der Reduktion bzw. des Absetzens, denn das unterscheidet sich erheblich von der Strategie, initial mit Glukokortikoiden zu beginnen und dann dabei einfach nur pragmatisch zu bleiben.

Die konsequente Therapieanpassung ist eng mit den QS 2 und 3 verbunden, und ein sehr wichtiger Aspekt ist auch dabei das T2T-Prinzip im Sinne der konsequenten Eskalation der Medikation, wenn die Remission noch nicht erreicht ist, aber erreicht werden kann. Dabei geht es allerdings aber nicht nur darum, sondern letztlich um jede Form der Therapieanpassung im Sinne eines personalisierten Managements, was neben differenzialdiagnostischen Einschätzungen und Abgrenzungen auch erforderliche Interventionen bei Funktionseinschränkungen, Komorbiditäten und psychosozialen Problemen umfasst (s. auch QS 5 und 6).

Beim Management von Notfällen geht es zunächst um die Definition – zum einen aus rein medizinischer Sicht und zum anderen aus Patient*innensicht, was sich erheblich unterscheiden kann. Kommt es zu Nervenausfallserscheinungen wegen einer atlantoaxialen Dislokation oder bei neu aufgetretenem Teerstuhl ist die Sache aus ärztlicher Sicht klar, in anderen Fällen hat die erbetene Akutkonsultation aber auch evtl. Zeit bis zur nächsten möglichen Sprechstunde innerhalb eines Werktages, weil der Praxisablauf sonst erschwert werden kann. Weil die akute oder subakute Gesundheitssituation aber am Telefon nicht immer ausreichend eingeschätzt werden kann, ist grundsätzlich eine geregelte Kooperation mit einer akutstationären Einrichtung erforderlich, denn diese hat ja 24 h/Tag „geöffnet“.

Das Management von Komorbidität ist auf der einen Seite Teil der rheumatologischen Versorgung, da die chronische Entzündung und auch die antirheumatische Medikation das Risiko für Komorbiditäten erhöhen können, auf der anderen Seite liegt es aber auch in der Verantwortung des Hausarztes. Zum einen sind die bei entzündlich rheumatischen Erkrankungen erhöhte Morbidität und Mortalität v. a. auf kardiovaskuläre Ereignisse zurückzuführen [1, 60] und die sowohl durch die Erkrankung als auch durch die Medikation bedingte erhöhte Inzidenz von Osteoporose und dadurch zustande gekommenen Frakturen mit einem erheblichen Leidensdruck der Betroffenen assoziiert [58]. Zum anderen gibt es in Deutschland eine Arbeitsteilung, die eigentlich den Hausärzt*innen die Verantwortung für etablierte kardiovaskuläre Risikofaktoren wie arterielle Hypertonie, Hypercholesterinämie, Diabetes und Rauchen zuweist. Hier sind gezielte Absprachen und gegenseitige Erinnerungen zielführend. Im Rahmen eines jährlichen Assessments können auch Rheumatolog*innen hier initiativ werden und zur Vorbeugung beitragen. Hinsichtlich der Osteoporoseprophylaxe ist eine frühzeitige Einleitung entsprechender Maßnahmen dagegen v. a. bei dem*r Rheumatolog*in als Verordner*in der Osteoporose-fördernden Medikation (Glukokortikoide) anzusiedeln.

Auch die Verordnung von nichtmedikamentösen Therapien wie regelmäßigem Funktionstraining und physiotherapeutischen Maßnahmen sowie die Motivation hin zu mehr körperlicher Aktivität gehören in diesen großen Bereich prophylaktischer und allgemein unterstützender Gesundheitsmaßnahmen.

Am 18.03.2021 wurde vom Gemeinsamen Bundesausschuss (G-BA) beschlossen, ein Disease-Management-Programm (DMP) für die RA in die DMP-Anforderungen-Richtlinien mit aufzunehmen. Dies ist neben der Entwicklung von QS ein guter Schritt in der Verbesserung der Versorgungsqualität der RA-Patient*innen, denn auch hier werden wichtige Schritte der Diagnostik und Therapie in Form von 10 Qualitätszielen definiert, welche sich in großen Teilen mit den hier veröffentlichten QS decken. In Ergänzung zu den hier veröffentlichen QS werden im DMP als Qualitätsziele gesondert nicht rauchende RA-Patient*innen und RA-Patient*innen mit arterieller Hypertonie und einem RR < 140/90 mm Hg aufgelistet [17].

Zusammengefasst handelt es sich bei den ersten 8 QS für rheumatologische Versorgung um einen ersten wichtigen Meilenstein in Richtung einer systematischen kontinuierlichen Verbesserung der Versorgungsqualität für Patient*innen mit RA in Deutschland. Diese QS fordern eine breite systemisch internistische Versorgung der*s Patient*in von Rheumatolog*innen, die der entzündlichen Erkrankung RA mit all ihren Folgen gerecht wird und sich nicht nur auf die Therapie der betroffenen Gelenke fokussiert. In weiteren Schritten wird zu prüfen sein, wie entsprechende Messungen implementiert und letztlich auch finanziert werden können.

## Supplementary Information





## Abkürzungen

Ag: Arbeitsgruppe
ASV: Ambulante spezialfachärztliche Versorgung
BDRh: Berufsverband Deutscher Rheumatologen
DGRh: Deutsche Gesellschaft für Rheumatologie
DMARD: Disease-modifying anti-rheumatic drug
EULAR: European League Against Rheumatism
LL: Leitlinien
NICE: National Institute for Health and Care
NRS: Numerical rating scale
QS: Qualitätsstandards
RA: Rheumatoide Arthritis
SLR: Systematische Literaturrecherche
VRA: Verband Rheumatologischer Akutkliniken
