# Supplementary material for: Development of quality standards for patients with rheumatoid arthritis for use in Germany
Source: Z Rheumatol. 2021 Oct 15;81(9):744–59. [Article in German] doi: 10.1007/s00393-021-01093-1 (PMC9646547; doi:10.1007/s00393-021-01093-1)
Supplement: Supplementary file 1 [file 393_2021_1093_MOESM1_ESM.pdf]

Zusatzmaterial 1: search terms zur SLR der Phase 1a

Identifikation von etablierten Qualitätsstandards für RA

<u>Suche 1 Medline (PubMed)</u>

1. arthritis, rheumatoid[mh]
2. (rheumatoid[tiab] OR reumatoid[tiab] OR rheumat*[tiab] OR reumat*[tiab]) AND (arthrit*[tiab] OR artrit*[tiab] OR diseas*[tiab] OR condition*[tiab] OR nodule*[tiab])
3. felty*[tiab] AND syndrome[tiab]
4. caplan*[tiab] AND syndrome[tiab]
5. #1 OR #2 OR #3 OR #4
6. Quality Indicators, Health Care[mh] OR healthcare quality indicators[tw] OR healthcare quality indicators[tiab] OR quality indicators[tiab]
7. #5 AND #6
8. #5 AND #6 Filters: Publication date from 2000/01/01 to 2020/12/31
9. #5 AND #6 Filters: Publication date from 2000/01/01 to 2020/12/31; English
10. #5 AND #6 Filters: Publication date from 2000/01/01 to 2020/12/31; English; German

148 Treffer

<u>Suche 2 Medline (PubMed)</u>

1. arthritis, rheumatoid[mh]
2. (rheumatoid[tiab] OR reumatoid[tiab] OR rheumat*[tiab] OR reumat*[tiab]) AND (arthrit*[tiab] OR artrit*[tiab] OR diseas*[tiab] OR condition*[tiab] OR nodule*[tiab])
3. felty*[tiab] AND syndrome[tiab]
4. caplan*[tiab] AND syndrome[tiab]
5. #1 OR #2 OR #3 OR #4
6. healthcare quality indicators[mh] OR healthcare quality indicators[tw] OR healthcare quality indicators[tiab]
7. #5 AND #6
8. #5 AND #6 Filters: Publication date from 2000/01/01 to 2020/12/31
9. #5 AND #6 Filters: Publication date from 2000/01/01 to 2020/12/31; English
10. #5 AND #6 Filters: Publication date from 2000/01/01 to 2020/12/31; English; German

121 Treffer

<u>Suche 1 Cochrane</u>

#1 MeSH descriptor: [Arthritis, Rheumatoid] explode all trees

#2 ((rheumatoid OR reumatoid OR rheumat* OR reumat*) AND (arthrit* OR artrit* OR diseas* OR condition* OR nodule*)):ti,ab,kw (Word variations have been searched)

#3 (felty* AND syndrome):ti,ab,kw (Word variations have been searched)

#4 (caplan* AND syndrome):ti,ab,kw (Word variations have been searched)

#5 #1 OR #2 OR #3 OR #4
#6 (healthcare quality indicators OR quality indicators):ti,ab,kw (Word variations have been searched)
#7 MeSH descriptor: [Quality Indicators, Health Care] explode all trees
#8 #6 OR #7
#9 #5 AND #8 with Cochrane Library publication date Between Jan 2000 and Dec 2020

399 Treffer

Suche 2 Cochrane
#1 MeSH descriptor: [Arthritis, Rheumatoid] explode all trees
#2 ((rheumatoid OR reumatoid OR rheumat* OR reumat*) AND (arthrit* OR artrit* OR diseas* OR condition* OR nodule*)):ti,ab,kw (Word variations have been searched)
#3 (felty* AND syndrome):ti,ab,kw (Word variations have been searched)
#4 (caplan* AND syndrome):ti,ab,kw (Word variations have been searched)
#5 #1 OR #2 OR #3 OR #4
#6 MeSH descriptor: [Quality Indicators, Health Care] explode all trees
#7 (healthcare quality indicators):ti,ab,kw (Word variations have been searched)
#8 #6 OR #7
#9 #5 AND #8 with Cochrane Library publication date Between Jan 2000 and Dec 2020

30 Treffer

Zusatzmaterial 2: search terms zur SLR der Phase 1 b

Identifikation von relevanten Versorgungslücken

Suche 1 Medline (PubMed)

1. arthritis, rheumatoid[mh]
2. (rheumatoid[tiab] OR reumatoid[tiab] OR rheumat*[tiab] OR reumat*[tiab]) AND (arthrit*[tiab] OR artrit*[tiab] OR diseas*[tiab] OR condition*[tiab] OR nodule*[tiab])
3. felty*[tiab] AND syndrome[tiab]
4. caplan*[tiab] AND syndrome[tiab]
5. #1 OR #2 OR #3 OR #4
6. patient care[mh] OR patient care[tiab] OR patient care[ti] OR care[tiab] OR care[ti]
7. #5 AND #6
8. gap[tiab] OR gap[ti] OR gap[tw] OR deficit[tiab] OR deficit[ti] OR deficit[tw] OR unmet need[tiab] OR unmet need[ti] OR unmet need[tw]
9. #7 AND #8
10. #7 AND #8 Filters: English
11. #7 AND #8 Filters: English; German
12. #7 AND #8 Filters: Humans; English; German

13. #7 AND #8 Filters: Publication date from 2005/05/01 to 2020/05/01; Humans; English; German

118 Treffer

<u>Suche 2 Medline (PubMed)</u>
1. arthritis, rheumatoid[mh]
2. (rheumatoid[tiab] OR reumatoid[tiab] OR rheumat*[tiab] OR reumat*[tiab]) AND (arthrit*[tiab] OR artrit*[tiab] OR diseas*[tiab] OR condition*[tiab] OR nodule*[tiab])
3. felty*[tiab] AND syndrome[tiab]
4. caplan*[tiab] AND syndrome[tiab]
5. #1 OR #2 OR #3 OR #4
6. patient care[mh] OR patient care[tiab] OR patient care[ti] OR care[tiab] OR care[ti] OR health care quality[mh] OR health care quality[ti] OR health care quality[tiab] OR Quality of Health Care[mh] OR Quality of Health Care[ti] OR Quality of Health Care[tiab] OR Ambulatory Care[mh] OR Ambulatory Care[tiab] OR Ambulatory Care[ti] OR Outcome and Process Assessment, Health Care[mh] OR Standard of Care[mh] OR Standard of Care[tiab] OR Standard of Care[ti] OR Patient Acceptance of Health Care[mh]
7. #5 AND #6
8. gap[tiab] OR gap[ti] OR gap[tw] OR deficit[tiab] OR deficit[ti] OR deficit[tw] OR unmet need[tiab] OR unmet need[ti] OR unmet need[tw]
9. #7 AND #8
10. #7 AND #8 Filters: Publication date from 2005/05/01 to 2020/05/01
11. #7 AND #8 Filters: Publication date from 2005/05/01 to 2020/05/01; English
12. #7 AND #8 Filters: Publication date from 2005/05/01 to 2020/05/01; Humans; English; German

325 Treffer

<u>Suche 3 Medline (PubMed)</u>
1. rheumatoid arthritis[mh]
2. gap[tiab] OR gap[ti] OR gap[tw] OR deficit[tiab] OR deficit[ti] OR deficit[tw] OR unmet need[tiab] OR unmet need[ti] OR unmet need[tw]
3. #1 AND #2
4. #1 AND #2 Filters: English
5. #1 AND #2 Filters: English; German
6. #1 AND #2: Filters: Humans; English; German
7. #1 AND #2: Filters: Publication date from 2005/05/01 to 2020/05/01; Humans; English; German

222 Treffer

<u>Suche 1 Cochrane</u>
1. MeSH descriptor: [Arthritis, Rheumatoid] explode all trees
2. (rheumatoid OR reumatoid OR rheumat* OR reumat*):ti,ab,kw (Word variations have been searched)
3. #1 OR #2
4. ((arthrit* OR artrit* OR diseas* OR condition* OR nodule*) OR (felty* AND syndrome) OR (caplan* AND syndrome)):ti,ab,kw (Word variations have been searched)

5. #3 AND #4
6. MeSH descriptor: [Patient Care] explode all trees
7. (patient care OR care):ti,ab,kw (Word variations have been searched)
8. #6 OR #7
9. (gap OR deficit OR unmet need):ti,ab,kw (Word variations have been searched)
10. #5 AND #8 AND #9 with Cochrane Library publication date Between May 2005 and May 2020, in Trials

43 Treffer

<u>Suche 2 Cochrane</u>
1. MeSH descriptor: [Arthritis, Rheumatoid] explode all trees
2. (rheumatoid OR reumatoid OR rheumat* OR reumat*):ti,ab,kw (Word variations have been searched)
3. #1 OR #2
4. ((arthrit* OR artrit* OR diseas* OR condition* OR nodule*) OR (felty* AND syndrome) OR (caplan* AND syndrome)):ti,ab,kw (Word variations have been searched)
5. #3 AND #4
6. MeSH descriptor: [Patient Care] explode all trees
7. (patient care OR care):ti,ab,kw (Word variations have been searched)
8. MeSH descriptor: [Quality of Health Care] explode all trees
9. (health care quality):ti,ab,kw (Word variations have been searched)
10. (Quality of Health Care):ti,ab,kw (Word variations have been searched)
11. MeSH descriptor: [Ambulatory Care] explode all trees
12. (Ambulatory Care):ti,ab,kw (Word variations have been searched)
13. MeSH descriptor: [Outcome and Process Assessment, Health Care] explode all trees
14. MeSH descriptor: [Standard of Care] explode all trees
15. (Standard of Care):ti,ab,kw (Word variations have been searched)
16. MeSH descriptor: [Patient Acceptance of Health Care] explode all trees
17. #6 OR #7 OR #8 OR #9 OR #10 OR #11 OR #12 OR #13 OR #14 OR #15 OR #16
18. (gap OR deficit OR unmet need):ti,ab,kw (Word variations have been searched)
19. #5 AND #17 AND #18 with Cochrane Library publication date Between May 2005 and May 2020, in Trials

59 Treffer

<u>Suche 3 Cochrane</u>
1. MeSH descriptor: [Arthritis, Rheumatoid] explode all trees
2. (gap OR deficit OR unmet need):ti,ab,kw (Word variations have been searched)
3. #1 AND #2 with Cochrane Library publication date Between May 2005 and May 2020, in Trials

22 Treffer
